# 

**Published:** 1862-12

**Authors:** 


New Series, Vol. 5, No. 12. Whole Series, Vol.

Dr Graham
Brownsburg Ind
Barker

### THE CHICAGO

### MEDICAL JOURNAL.

DANIEL BRAINARD, M. D.,
J. ADAMS ALLEN, M. D.,
Editors and Proprietors.

DECEMBER, 1862.

CHICAGO:

WM. PIGOTT, BOOK AND JOB PRINTER, 130 CLARK STREET.

1862.

